# Modulation of Insulin Sensitivity by Insulin-Degrading Enzyme

**DOI:** 10.3390/biomedicines9010086

**Published:** 2021-01-17

**Authors:** Carlos M. González-Casimiro, Beatriz Merino, Elena Casanueva-Álvarez, Tamara Postigo-Casado, Patricia Cámara-Torres, Cristina M. Fernández-Díaz, Malcolm A. Leissring, Irene Cózar-Castellano, Germán Perdomo

**Affiliations:** 1 Instituto de Biología y Genética Molecular, University of Valladolid-CSIC, 47003 Valladolid, Spain; carlosmanuel.gonzalez.casimiro@uva.es (C.M.G.-C.); bmerino@ibgm.uva.es (B.M.); elenacasanueva15@gmail.com (E.C.-Á.); tamara.postigo@uva.es (T.P.-C.); patriciacamaratorres1@gmail.com (P.C.-T.); 2 Molecular Oncology Group, IMDEA Food Institute, CEI UAM + CSIC, 28049 Madrid, Spain; cristinamaria.fernandez@imdea.org; 3 Institute for Memory Impairments and Neurological Disorders, University of California, Irvine (UCI MIND), Irvine, CA 92697, USA; m.leissring@uci.edu; 4 Centro de Investigación Biomédica en Red de Diabetes y Enfermedades Metabólicas Asociadas (CIBERDEM), 28029 Madrid, Spain

**Keywords:** insulin-degrading enzyme, insulin resistance, pancreas, liver, insulin receptor, glucose transporters

## Abstract

Insulin-degrading enzyme (IDE) is a highly conserved and ubiquitously expressed metalloprotease that degrades insulin and several other intermediate-size peptides. For many decades, IDE had been assumed to be involved primarily in hepatic insulin clearance, a key process that regulates availability of circulating insulin levels for peripheral tissues. Emerging evidence, however, suggests that IDE has several other important physiological functions relevant to glucose and insulin homeostasis, including the regulation of insulin secretion from pancreatic β-cells. Investigation of mice with tissue-specific genetic deletion of *Ide* in the liver and pancreatic β-cells (L-IDE-KO and B-IDE-KO mice, respectively) has revealed additional roles for IDE in the regulation of hepatic insulin action and sensitivity. In this review, we discuss current knowledge about IDE’s function as a regulator of insulin secretion and hepatic insulin sensitivity, both evaluating the classical view of IDE as an insulin protease and also exploring evidence for several non-proteolytic functions. Insulin proteostasis and insulin sensitivity have both been highlighted as targets controlling blood sugar levels in type 2 diabetes, so a clearer understanding the physiological functions of IDE in pancreas and liver could led to the development of novel therapeutics for the treatment of this disease.

## 1. Introduction

Insulin-degrading enzyme (IDE; EC 3.4.24.56; a.k.a. insulin protease, insulinase, insulysin, insulin-glucagon protease, neutral thiol protease, metalloendoprotease, amyloid-degrading protease or peroxisomal protease) is a neutral Zn^2+^ metallo-endopeptidase that is ubiquitously expressed in insulin-responsive and non-responsive cells [1,2]. IDE belongs to a distinct superfamily of zinc-metalloproteases (clan M16) sometimes called “inverzincins” because they are characterized by a zinc-binding consensus sequence (HxxEH) that is inverted with respect to the sequence in most conventional metalloprotease (HExxH) [1,3,4]. IDE and its homologues represent an interesting example of convergent evolution; despite independent origins, IDE shares striking structural and functional similarity with conventional metalloproteases (clan M13 family; e.g., thermolysin and neprilysin) [3,4,5].

IDE and its homologs and paralogs are highly conserved and present in phylogenetically diverse organisms, ranging from viruses to humans [6], highlighting the fact that IDE is a multifunctional protein with proteolytic and non-proteolytic functions (Table 1; Table 2). Human IDE shares significant sequence similarity with orthologs in bacteria and yeast, for example, including the HxxEH zinc-binding motif. The protease has insulin-binding and -degrading activity in *Neurospora crassa*, *Acinetobacter calcoaceticus* and *Escherichia coli* (*E. coli* protease III or pitrilysin) [7,8,9,10]. IDE orthologs exhibit a periplasmic localization in *E. coli* [11] and *A. calcoaceticus* [9], whereas the ortholog in *N. crassa* is membrane bound, leading to a proposed function as a putative insulin receptor (IR) [7]. Of interest, the yeast IDE homologues Axl1p and Ste23 are incapable of degrading insulin despite possessing the conserved zinc-binding motif [12]. The proteolytic activity of Ste23 is required, however, for N-terminal cleavage of the pro-a-factor, the precursor of a pheromone (a-factor) involved in the mating response of haploid yeast cells [13]. Interestingly, rat IDE can promote the formation of mature a-factor in vivo, suggesting that the functional conservation of IDE, Axl1p and Ste23 may be not be bidirectionally conserved [12]. Humans and other mammals also express several IDE paralogs (Table 2), including N-arginine dibasic convertase (a.k.a., nardilysin), a cytosolic, secreted and membrane bound peptidase [14]. Of relevance to the present review, nardilysin regulates β-cell function and identity through the transcriptional factor MafA [15] and also prevents the development of diet-induced steatohepatitis and liver fibrogenesis by regulating chronic liver inflammation [16]. Nardilysin has several other roles unrelated to hepatic or pancreatic function, however, such as modulation of thermoregulation [17]. It also mediates cell migration by acting as a specific receptor for heparin-binding epidermal growth factor-like growth [18]. This protein provides a good example of how paralogs (rather than orthologs) can evolve to develop diverse functions.

### 1.1. The Discovery of IDE: An Historical Perspective

More than 70 years ago, Mirsky and Broh–Kahn characterized the existence of a proteolytic activity they named insulinase, which inactivated insulin in rat tissue extracts from liver, kidney and muscle in vitro [19]. The insulinase activity was a mixture of specific and non-specific proteases. Almost four decades of work, within several laboratories, would be conducted on this crude activity before Roth and colleagues eventually cloned the cDNA of *Ide*, revealing that the insulin-degrading activity within these extracts was attributable primarily if not exclusively to the action of just one protease, IDE [20,21]. Work on this extract within the early literature, however, resulted in several notable findings. For instance, Mirsky reported that fasting markedly reduced insulinase activity of rat liver extracts as compared with fasted rats subsequently fed a regular diet, suggesting a relationship between the insulin content of pancreas and hepatic insulinase activity [22]. However, refeeding of fasted rats with a high-carbohydrate diet resulted in a greater increase in insulinase than refeeding with a high-fat diet (HFD) [23]. Interestingly, Mirsky and colleagues were the first to report the existence of a substance that, in vitro, inhibited the insulinase activity in liver extracts, and they also demonstrated the effect of crude insulinase-inhibitor preparations on insulin degradation in vivo [24,25,26]. Identification of endogenous proteins that interact with and modulate IDE function has been only partially successful. For example, Brush and colleagues partially purified four competitive IDE inhibitors from human serum, whose molecular weights were in a range that includes insulin (Cohn fraction IV) [27]. Ogawa and colleagues purified an endogenous ~14-kDa protein from rat liver that in a competitive manner inhibited insulin binding and insulin degradation by IDE [28]. Other groups partially purified additional competitive [29] and noncompetitive [30] insulinase inhibitors from rat tissues, but their identities remain unknown. However, Saric and colleagues successfully identified ubiquitin as an IDE-interacting protein that inhibited the proteolytic activity of IDE in a reversible, ATP-independent manner [31]. Although the physiological relevance of the non-covalent, and energy-independent interaction between IDE and ubiquitin remains to be established, these findings may have alternative implications, such as the possibility that IDE interacts with ubiquitin-like modifiers. Finally, Mirsky described that the hypoglycemic effect of oral administration of sulfonylureas in diabetic patients was associated with non-competitive inhibition of insulinase in liver [32], leading to speculation that the mechanism of action of sulfonylureas was inhibition of insulin degradation [33].

The pioneering investigation of the insulinase led to the formulation of an innovative concept to explain the etiology of diabetes. At a time when the classical view of diabetes, proposed by von Mehring and Minkowski as well as Banting and Best [34,35], was a decrease in the production of insulin by pancreatic β-cells, Mirsky hypothesized that an increase in the rate of insulin degradation by extrapancreatic tissues, could explain the insulin insufficiency in some diabetic patients [36]. The role of IDE on the pathophysiology of diabetes has evolved over time, and it will be discussed in this review.

### 1.2. The Function of IDE as a Protease of Insulin

IDE was first characterized by its capacity to degrade insulin into several fragments in vitro*,* yielding major and minor products. The initial cleavage events occur at the middle of the insulin A and B chains, without specific amino acid requirements, suggesting that substrate recognition by IDE depends on tertiary structure rather than primary amino acid sequence [37,38,39,40]. Consistent with this, whereas IDE has a high affinity for insulin (K_m_ ~0.1 µM), proinsulin is a poor substrate that is hydrolyzed at very slow rates, acting as a competitive inhibitor [41,42]. Likewise, insulin-like growth factor I (IGF-I) and IGF-II are substrates of IDE, being IGF-II degraded more rapidly than IGF-I, but both acting as competitive inhibitors of IDE [42]. Another potent inhibitor of the insulin-degrading activity of IDE is the leader peptide of rat prethiolase B (P27 peptide), present in peroxisomes [43].

For decades, the standard assay for assessing insulin-protease activity was the use of purified or partially purified enzyme preparations of IDE (e.g., from erythrocytes) and [^125^I]-labelled insulin. Two methods have been used to quantify the degradation of these radiolabeled peptides, the trichloroacetic acid (TCA) precipitation assay and high-performance liquid chromatography (HPLC), the former being the most sensitive [44,45]. Several other IDE activity assays have been developed, such as fluorogenic FRET-based peptide substrates derived from the sequence of bradykinin [46] or other IDE substrates [47]. However, IDE appears to process such short peptides markedly differently from intermediate-sized substrates [48]; for instance, the hydrolysis of such substrates is activated by ATP [49] and other nucleoside polyphosphates [50], certain small molecules [51] and several substrates [52], while these compounds have no effect or actually inhibit the degradation of more physiological substrates. To account for this intriguing substrate specificity of IDE, several substrate-specific degradation assays have been developed for different IDE substrates, including amyloid β-protein (Aβ) [53], glucagon [54] and amylin [55]. Unfortunately, similarly facile assays for insulin degradation have not yet been identified, so insulin degradation is now typically quantified by ELISA or HPLC.

**Figure 1:**
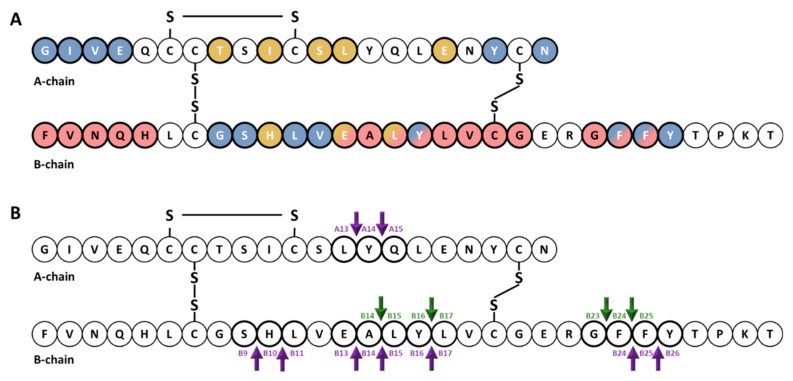
Cartoon illustrating the primary structure and cleavage products of human insulin. (**A**) primary structure of insulin showing amino acids that interact with IDE (red color) [63,64,65] and with site 1 (blue color) and site 2 (gold color) of the IR [66,67,68,69]. (**B**) cleavage products generated by endosomal proteases. At an early time, following insulin endocytosis, endosomal proteases account for major degradation products containing an intact A-chain, and cleavages in the B-chain (green arrows). Purple arrows indicate IDE cleavage sites effected by IDE in vitro [39].

Early studies of the kinetics of insulin degradation suggested that the endosomal apparatus is a physiological site of degradation of internalized insulin in hepatocytes [56,57,58]. Within the acidic endosomal lumen, the insulin-IR complex dissociates and insulin is degraded by endosomal proteases, whereas the IR is recycled to the plasma membrane of the hepatocyte [38]. Over the past several decades, it has been proposed that endosomal degradation of insulin is mediated by the action of three endosomal proteases: Cathepsin D [59], neutral Arg aminopeptidase [60] and IDE [38]. Cathepsin D has been shown to be responsible for the majority of the proteolytic degradation within endosomes, in general [59,61]. Neutral Arg aminopeptidase is an endosomal Arg convertase involved in the removal of Arg residues from internalized monoarginyl insulin prior endosomal acidification [60]. The action of this protease is highly selective towards [ArgA0]-human insulin peptide, a proinsulin intermediate containing an additional Arg at the amino-terminal insulin A-chain [60]. The role of IDE in endosomal proteolysis of internalized insulin, however, remains controversial, even though many of the primary sites of cleavage of internalized insulin are consistent with those produced by purified IDE [43,61,62]. At an early time, following insulin endocytosis, endosomal proteases account for major degradation products containing an intact A-chain, and cleavages in the B-chain at the Phe^B24^-Phe^B25^, Gly^B23^-Phe^B24^, Tyr^B16^-Leu^B17^ and Ala^B14^-Leu^B15^ peptide bonds [56,57,58] (Figure 1). At a later stage, insulin is degraded to its constituent amino acids within endosomes and/or lysosomes [38,58].

The role of IDE as a protease of insulin has been demonstrated over time in cell extracts and intact cells. Overexpression of human IDE (h*Ide*) in Chinese hamster ovary (CHO) cells [70] and monkey kidney COS cells [71] enhanced the extracellular degradation of exogenously applied insulin. Likewise, cell extracts from NIH3T3 cells overexpressing *Drosphila* IDE (d*Ide*) exhibited enhanced insulin-degrading activity [72]. Based on the use of lysosomotropic agents (which prevent acidification of intracellular lysosomes and endosomes), Kuo and colleagues showed that insulin degradation was affected by intact cells via a d*Ide*-mediated intracellular pathway, independently of the lysosome [71]. In addition, this same team created a stably transfected Ltk^−^ cell line with dexamethasone-inducible overexpression of h*Ide*. IDE expression upon glucocorticoid induction resulted in increased IDE insulin degradation in both cell lysates and intact cells [73].

Consistent with these findings, non-specific inhibitors of IDE decreased intracellular insulin degradation in intact HepG2 cells [74], rat L6 myoblasts [75], and mouse BC3H1 muscle cells [76]. Similarly, monoclonal antibodies against IDE almost completely abolished the insulin-degrading activity of IDE from erythrocytes, and microinjection of these antibodies into HepG2 cells reduced intracellular [^125^I]-insulin degradation by ~50% [77]. Furthermore, insulin degradation was diminished by ~50% in HepG2 transfected cells with siRNA against h*Ide*, in parallel with a reduction by more than 50% in IDE protein and mRNA levels [78]. Similarly, CRISPR/Cas9 targeting of *Ide* in CHO cells abolished insulin degradation [79]. Finally, cytosolic and membrane fractions of liver from mice with homozygous deletion of the *Ide* gene (IDE-KO mouse) showed impaired [^125^I]-insulin degradation [80]. Taken together, the above-mentioned studies indicated that increasing IDE activity increases cellular insulin degradation, and conversely decreasing its activity reduces insulin degradation.

Despite the large body of evidence supporting a role for IDE in insulin degradation within cultured cells, IDE resides primarily in the cytosol and does not have a signal peptide, so precisely where IDE interacts with insulin or other substrates remains an unresolved question. On the one hand, Zhao and colleagues found that IDE is exported via an unconventional protein secretion pathway [81]. On the other hand, a recent study by Song and colleagues [82] found only very low levels of IDE secretion from HEK293 and BV2 cells, in quantities never exceeding the non-specific release of other cytosolic proteins such as lactate dehydrogenase. 

The involvement of IDE in insulin degradation in vivo is similarly controversial. For instance, on the one hand, Farris and colleagues [80], and Abdul–Hay and colleagues [83] found that IDE-KO mice exhibited significant increases in plasma insulin levels. On the other hand, Miller and colleagues [84] and Steneberg and colleagues [85] reported that plasma insulin levels in IDE-KO mice were unchanged relative to wildtype controls. Moreover, we have shown that mice with liver-specific deletion of *Ide* (L-IDE-KO mice) display normal insulin levels when fed a regular diet [86], but elevated levels when fed a HFD [87], as is discussed in greater detail below.

### 1.3. Other Proteolytic Functions of IDE

IDE can degrade several other substrates with lower affinity than insulin, including glucagon [88], somatostatin [89], amylin [90], Aβ [91], amyloid precursor protein intracellular domain [92], amyloid Bri and amyloid Dan [93], atrial natriuretic peptide [94], bradykinin and kallidin [95,96], calcitonin and β-endorphin [5], growth hormone-release factor [97], transforming growth factor-α [98], oxidized hemoglobin [99], cytochrome c [100], chemokine ligand (CCL)3 and CCL4 [101] and HIV-p6 protein [102].

A role for IDE in the processing of insulin epitopes for helper T cells has been reported by Semple and colleagues [103,104]. IDE degrades human insulin into peptides that are presented by murine TA3 B-cell antigen-presenting cells to HI/I-Ad-reactive T cells [103]. Of note, IDE is necessary but not sufficient for the recognition of insulin by T cells [103,104]. More controversial is the role of IDE in the proteasome-independent processing of peptides. As shown by Parmentier and colleagues, MAGE-A3, a cytosolic human tumor protein, is degraded by IDE, leading to different sets of antigenic peptides presented by major histocompatibility complex (MHC) class I molecules to cytotoxic T lymphocytes (CTLs) [105]. Immunodepletion of IDE abolished the capacity to produce the antigenic peptide (MAGE-A3_168–176_), whereas expression of recombinant human IDE was able to produce the antigenic peptide [105]. In addition, *Ide* RNAi-treated cells reduced the ability of CTLs to recognize tumor cells [105]. On the other hand, Culina and colleagues, using a number of MHC-I class molecules and a loss-of-IDE-function approach in human cell lines and two different mouse strains (IDE-KO mouse and IDE-KO mouse back-crossed to the non-obese diabetic (NOD) strain), concluded that IDE does not play a general major role in peptide loading to MHC-I molecules [106].

Other non-insulin-related proteolytic functions of IDE include degrading cleaved leader peptides of peroxisomal proteins targeted by the type II motif [43] and, possibly, cleaved mitochondrial targeting sequences, in this case by a mitochondrial form of IDE generated by alternative translation initiation [107]. IDE has also been implicated in the formation and/or degradation of “cryptic” peptides (i.e., hidden peptides derived from proteolytic processing of a substrate with different biochemical functions of parent protein), which is the case for IDE-mediated regulation of cryptic peptides from the neuropeptide FF (NPFF) precursor (pro-NPFF_A_) [108] as well as Aβ [109]. In addition, IDE has been proposed to mediate the degradation of nociceptin/orphanin 1–16 (OFQ/N), a class of neuropeptides involved in pain transmission. Interestingly, the main hydrolytic peptides of OFQ/N produced by IDE, but not the neuropeptide itself, exhibited inhibitory activity towards IDE-mediated degradation of insulin [110].

An example of multiple catalytic and non-catalytic functions of IDE is its role in binding and degrading viral proteins. For instance, Li and colleagues showed that IDE interacts with the glycoprotein E (gE) from varicella zoster virus (VZV) and proposed that IDE is the cellular receptor for the virus [111]. This group subsequently demonstrated that binding of IDE to the N-terminal domain of gE produced a conformational change, increasing its susceptibility to proteolysis [112]. Berarducci and colleagues found that gE/IDE interaction contributed to skin virulence in vivo [113]. In contrast, the gE/IDE interaction was not necessary for VZV infection of T cells in vivo [113]. On the other hand, IDE is necessary and sufficient for degradation of the mature p6 protein of the human immunodeficiency virus 1 (HIV-1) [102,114]. Of note, p6 is degraded 100-fold more efficiently than insulin [102]. Virus replication was reduced by exogenous insulin or pharmacological inactivation of IDE with the inhibitor 6bK [102,115].

### 1.4. Non-Proteolytic Functions of IDE

IDE has been reported to directly interact with androgen and glucocorticoid receptors, enhancing specific DNA binding of both receptors [116]. Non-competitive inhibition of IDE’s catalytic activity did not block the binding of the androgen receptor to IDE, but competitive inhibition of IDE blocked its binding, suggesting that IDE-binding sites for the receptor and insulin are identical or overlapping [116]. Interestingly, dexamethasone, a synthetic glucocorticoid, significantly reduced insulin binding to IDE without affecting expression levels of the protease in rat hepatoma cells, most likely by inducing a conformational change or blocking insulin-binding sites [117]. Conversely, the steroid’s effect was blocked by insulin [117]. The interaction of IDE with androgen and glucocorticoid receptors may be of relevance for steroid hormone action and metabolism, the crosstalk between insulin and steroid hormones, and the pathophysiology of glucocorticoid-mediated insulin resistance [118].

Intriguingly, IDE co-localizes with the ~50-amino acid cytoplasmic tail of the scavenger receptor type A (SR-A), an important domain for SR-A function, in mouse macrophages [119]. The biological significance of this interaction is uncertain, because IDE deficiency in bone-marrow derived macrophages (BMDMs) did not alter protein levels of SR-A or its ability to uptake low-density lipoprotein (LDL)-cholesterol, albeit its deficiency in these cells was associated with higher levels of intermediate density lipoproteins, LDL-cholesterol and accelerated atherogenesis in LDL receptor knockout (*Ldlr*^−/−^) mice [119]. Considering that SR-A participates in multiple cellular processes, including regulation of inflammatory cytokine synthesis through its interaction with TLR4, it is tempting to hypothesize that IDE deficiency in macrophages may cause an inflammatory milieu surrounding the arterial wall, thus contributing to the pathophysiology of atherogenesis.

IDE co-immunoprecipitates with SIRT4, a protein with no histone deacetylase activity but with associated ADP-ribosyl-transferase activity that resides in the mitochondrial matrix [120]. SIRT4 expression is detected in several mouse tissues including liver [121,122], and human pancreatic β-cells [120,122]. SIRT4 depletion in INS1 and MIN6 cells markedly increased insulin secretion without altering basal secretion and intracellular insulin content [120,122]. Conversely, SIRT4 overexpression in INS1 cells suppressed glucose-induced insulin secretion. Of note, insulin secretion stimulated with the secretagogue KCl, which bypasses mitochondrial activation, remained unaltered in SIRT4-depleted INS-1E cells [120]. Once more, the biological significance of this interaction remains undeciphered, because there are no reports on the ADP-ribosylation of IDE, and the mitochondrial function of IDE is not clarified. However, IDE depletion in INS1-E cells using siRNA-IDE and shRNA-IDE significantly decreases glucose-stimulated insulin secretion [123]. The contributions of the cytosolic and mitochondrial forms of IDE in regulating insulin secretion, and the association of the later with SIRT4, warrants further research.

IDE has been identified as an interacting partner of intermediate filaments, one of the three major cytoskeletal components that serve as a scaffold for signaling molecules, modulating their distribution and activity [124]. Specifically, IDE has been shown to interact with disassembled and soluble vimentin/nestin complexes during mitosis [125]. Vimentin plays a dominant role in targeting IDE to the complex and binds to IDE with higher affinity than nestin. Phosphorylation of vimentin is not required for its binding to IDE, but the interaction is enhanced by vimentin phosphorylation at Ser-55. On the other hand, binding of IDE to nestin promotes the disassembly of vimentin intermediate filaments, most likely by rendering the phosphorylated vimentin more accessible for IDE. The binding of IDE to nestin is phosphorylation independent. Interestingly, the binding of nestin or phosphorylated vimentin suppressed by ~2-fold the insulin-degrading activity of IDE but increased its proteolytic activity toward bradykinin [125]. These data suggest that IDE may be involved in regulating the turnover and/or subcellular localization of cytosolic proteins and peptides. In this context, it has to be noted that integrins are a major family of cell adhesion receptors that mediate attachment of cells to the extracellular matrix. Recruitment of integrins and other proteins forms multi-protein complexes on the cytoplasmic face of the membrane named focal adhesions, which allow the anchoring of the actin cytoskeleton to the plasma membrane, providing a linkage between the extracellular environment and the cytoplasm. Integrins do not have intrinsic kinase activity and signaling depends upon the recruitment and activation of focal adhesion kinase (FaK), a cytoplasmic protein tyrosine kinase [126,127]. Significantly, Liu and colleagues identified IDE as a binding partner that interacts with C-terminal domain of FaK [128]. The relevance of the interaction between IDE and FaK in regulating recruitment of cytoskeletal proteins and the assembly of focal adhesions, a process that is important for cell migration, survival and proliferation, remains to be elucidated.

IDE, in a non-proteolytic manner, binds to α-synuclein oligomers leading to the formation of stable and irreversible complexes, precluding amyloid formation [129,130]. α-synuclein is a synaptic signaling protein with three domains—the N-terminus, which interacts with membranes, the amyloidogenic domain and the C-terminus, which is involved in the pathogenesis of Parkinson’s disease [131]. Interestingly, the catalytic activity of IDE on a bradykinin-based fluorogenic substrate was increased in the presence of α-synuclein [129]. The interaction between both proteins appears to require electrostatic attraction involving the exosite region of IDE, which is positively charged, and the C-terminus of α-synuclein, which contains many negatively charged amino acids [130]. The role of IDE in the turnover of amyloidogenic proteins and the non-proteolytic prevention of toxic amyloid formation—in the case of α-synuclein via a so-called “dead-end chaperone function”—appears to be important for pancreatic β-cells function. In this connection, Steneberg and colleagues showed that genetic deletion of *Ide* in pancreatic β-cells led to the formation α-synuclein oligomers and fibril accumulation, which was associated with impaired insulin secretion and reduced granule turnover, possibly by disruption of the microtubule network [85].

Sorting nexin 5 (SNX5) is a member of the sorting nexin family that regulates intracellular trafficking and is abundantly expressed in kidney [132,133]. Its expression is reduced in Zucker rats, a model of obesity, hyperinsulinemia and insulin resistance [134]. The *Snx5* gene is located on chromosome 20p, a susceptibility quantitative trait locus for high fasting plasma insulin levels and insulin resistance [135]. Li and colleagues have shown that IDE colocalizes with SNX5 in the brush border membrane of proximal tubules and the luminal side of distal convoluted tubules of human and rat kidneys, in addition to the plasma membrane and perinuclear area of human renal proximal tubule cells (hRPTCs) [134]. Furthermore, exposure of hRPTCs to insulin increased colocalization and co-immunoprecipitation of IDE and SNX5. Interestingly, SNX5-depleted hRPTCs exhibit reduced IDE activity and protein levels, in parallel with decreased expression of the IR and downstream insulin signaling [134,136]. Similarly, renal subcapsular infusion of SNX5-specific siRNA decreased IDE mRNA and protein expression in kidneys of mice [134]. These studies underline the potential significance of renal IDE in the regulation of circulating insulin levels as well as insulin sensitivity in kidneys.

The human retinoblastoma (RB) protein acts as a tumor suppressor that negatively regulates cell cycle progression at the G1/S transition through its interaction with the E2F family of transcription factors [137]. IDE co-purifies with RB on proteasomal preparations of breast cancer and hepatoma cells [138]. Similarly, IDE co-immunoprecipitates with the tumor suppressor phosphatase and tensin homolog deleted on chromosome 10 (PTEN) [139]. IDE accelerates PTEN degradation by SIRT4 in response to nutritional starvation stresses [139]. Although the underlying molecular mechanisms have not been fully elucidated, these findings support a role for IDE in insulin-driven oncogenesis. Likewise, Tundo and colleagues have hypothesized that IDE, in a heat shock protein-like fashion, may be implicated in cell growth regulation and cancer progression [140]. In normal cells (human fibroblasts cell line and human peripheral blood lymphocytes) and malignant cells (human neuroblastoma cell line (SHSY5Y) and human lymphoblastic-like cells line (Jurkat cells)) exposed to heat shock, H_2_O_2_ and serum starvation, IDE is markedly up-regulated at both protein and mRNA levels. Additionally, delivery of IDE siRNA to SHSY5Y cells led to extensive apoptotic cell death; and administration of ATRA (a vitamin A precursor used in the clinical treatment of neuroblastoma) significantly decreased intracellular IDE content [140].

### 1.5. Molecular and Biochemical Characteristics of IDE

IDE is synthesized as a single polypeptide with a molecular weight of ~110 kDa by a gene located on human chromosome 10 q23–q25, and mouse chromosome 19, respectively [20,70]. *Ide* coding mutations have been associated with the development of T2DM in the Goto–Kakizaki rat model [141]. Fakhrai–Rad and colleagues identified two missense mutations (H18R and A890V) in IDE that decrease the ability to degrade insulin by 31% in transfected cells. They reported a synergistic effect of the two mutations on insulin degradation, which is somewhat puzzling given that H18R is present within the mitochondrial targeting sequence of IDE produced by alternative translation initiation [107]. Farris and colleagues, by contrast, found that recombinant IDE containing the A980V mutation alone exhibited reduced catalytic efficiency of both insulin and Aβ degradation, suggesting that this mutation is most relevant to proteolytic function [142]. Interestingly, no effect on insulin degradation was seen in cell lysates of *Ide*-transfected COS cells, suggesting that the effect may be dependent upon receptor-mediated internalization of the hormone [141,142].

IDE assembles as a stable homodimer, although it can exist as an equilibrium of monomers, dimers and tetramers [52]. Each monomer is comprised four homologous domains (named 1–4). The first two domains constitute the N-terminal portion (IDE-N), and the last two the C-terminal portion (IDE-C). IDE-N and IDE-C are joined by an extended loop of 28 amino acids. In the human IDE dimer, the interface between the two monomers is formed by 18 residues of domains 3 and 4 (IDE-C) [143]. Interestingly, the crystal structure of rat IDE, obtained by Hersh and colleagues [144], revealed a different homodimer interface than human IDE. In rat IDE, deletion of the last 18 amino acids abolished homodimer formation while simultaneously eliminating allosteric effects reflective of inter-subunit cooperativity [144]. The active site of IDE consists of a catalytic tetramer, HxxEHx_76_E, located inside domain 1, in which two histidine residues (H108 and H112) and a glutamate (E189) coordinate the binding of the Zn^2+^ ion and a second glutamate (E111) plays an essential role in catalysis. Glutamate E111 activates a catalytic water molecule for the nucleophilic attack that mediates peptide hydrolysis [63,145]. Although the catalytic site is entirely inside IDE-N domain, the IDE-C is necessary for correct substrate recognition [146]. Site-directed mutagenesis revealed that mutating IDE H108 (i.e., H108L and H108Q) abolished catalytic activity of the enzyme, but not the ability to bind insulin. Similarly, mutation E111Q abolished proteolytic activity [145,147].

**Figure 2:**
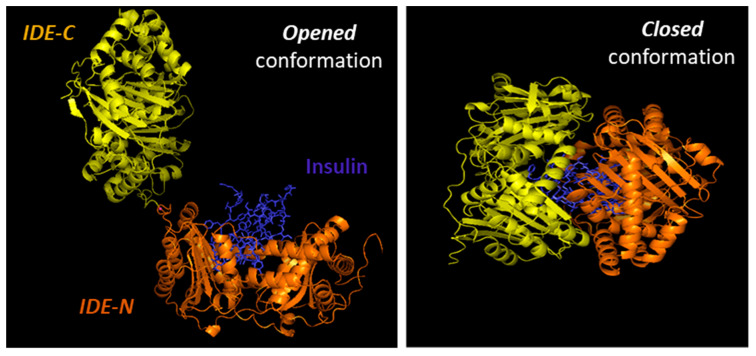
Cartoon illustrating the binding of insulin by IDE. IDE is in an equilibrium between “opened” and “closed” conformational states. In the absence of a substrate (e.g., insulin), IDE is preferentially in the closed conformation. IDE must adopt the open conformation for substrates to enter the internal chamber, whereas the protease must assume the closed conformation for proteolysis to occur. Release of the cleavage products requires a return to the open conformation.

Substrates for IDE are almost exclusively intermediate-size (~20–40 amino acids) peptide substrates, with rare exceptions, such as oxidized hemoglobin [99]. This size preference is the result of the overall structure of IDE, which resembles a clamshell, with two bowl-shaped domains (IDE-C and IDE-N) facing one another, connected by a hinge, and together forming a ~13,000-Å^3^ internal chamber (Figure 2). These domains can pivot on the hinge, thus adopting “open” and “closed” conformations. Transition to the open conformation is required for entry of substrates and exit of proteolytic products, and there is strong evidence that transition to the closed conformation is a requirement for proteolytic processing. Consistent with this, site-directed mutagenesis revealed that the complete active site of IDE is in fact bipartite, consisting of residues within both IDE-N and IDE-C [148], a conclusion that is confirmed by numerous crystal structures [149]. Due to the placement of the bipartite active site within the chamber of the closed protease, substrates must be small enough to fit completely within this chamber to be processed. To facilitate binding and subsequent cleavages at the catalytic site, larger substrates interact with an exosite within domain 2 located ~30 Å away from the active-site Zn^2+^, which anchors the N-terminus of several substrates [50]. Because of the unusual requirement that substrates fit within the internal chamber, the substrate selectivity of IDE is based more on the tertiary structure of substrates than their primary amino acid sequence. IDE shows some preference for cleavage at basic or bulky hydrophobic residues at the P1’site of the target protein [62], but this subsite specificity is not strict, and IDE commonly cleaves at vicinal peptide bonds within substrates [53]. Of note, substrates containing positively charged residues at their C-terminus are poor IDE substrates [63]. Thus glucagon, which lack of positively charged amino acids at the C-terminal is an IDE substrate, but not glucagon-like peptide [63].

The requirement for a transition between the “closed” and “open” conformations has an additional implication for the activity of IDE. There is extensive hydrogen bonding between the two halves of IDE, creating a “latch” that tends to maintain the protease in the closed conformation [63,150,151]. Consistent with this idea, most crystal structures of IDE, whether empty or occupied by substrate, show the protease in the closed conformation [63]. Notably, mutation of some of the residues mediating this interaction has been shown to activate the protease by as much as 15-fold [63]. It is estimated that in the absence of other factors, ~99% of IDE molecules are normally in the closed conformation (M.L. unpublished observations), suggesting that a significant of latent IDE activity could be untapped, for example, by compounds that disrupt this “latch” [51].

Interestingly, somatostatin, a hormone produced and secreted by the hypothalamus and in the pancreas by δ-cells, that inhibits glucose-stimulated insulin secretion [152], in addition to being a substrate of IDE, also regulates its function. Somatostatin binds to two additional exosites named “somatostatin-binding exosites” which play different roles according to the size of the substrates and its binding mode to the IDE catalytic cleft [95].

As mentioned above, a mitochondrial isoform of IDE was identified by Leissring and colleagues, which is formed by alternative translation initiation [107]. The open reading frames of human, rat, and mouse *Ide* cDNAs contain two in-frame translational codons encoding proteins beginning either at the first (Met^1^-IDE) or the 42nd amino acid (Met^42^-IDE). Met^42^-IDE (the shorter isoform) is the predominant isoform expressed in tissues and culture cells [107], because the nucleotide sequence surrounding the second initiation codon contains a better Kozak consensus sequence for initiation of translation. Although the Met^1^-IDE isoform is predicted to be less efficiently translated, it could nevertheless account for a significant fraction of total cellular IDE [107]. Currently, it is uncertain whether the mitochondrial isoform plays a major role in human disease.

In addition to the two possible translation initiation sites (Met^1^-IDE and Met^42^-IDE), Farris and colleagues identified a novel splice isoform in which exon 15a is replaced by a novel exon, 15b [153]. The resultant variant is widely expressed and present in both cytosol and mitochondria. The 15b-IDE isoform can exist as homodimer or as heterodimer with the 15a isoform. The catalytic efficiency of the 15b-IDE isoform is significantly lower than the 15a-IDE isoform [153].

IDE expression is regulated during cell differentiation and growth. During rat development (6–7 days of age) to adulthood, *Ide* mRNA levels increased in brain, testis and tongue with a concomitant decreased expression in muscle and skin but remained unchanged in other tissues. In the adult rat, *Id*e mRNA is higher in testis, tongue and brain, and lower in spleen, lung, thymus and uterus [2,154]. Interestingly, IDE activity is affected by aging. The highest IDE activity is observed in muscle, liver and kidneys of 4-week-old rats. The IDE activity in muscle and liver at 7 weeks of age is lower than at 4 weeks, with similar activity in kidney. The lowest activity of IDE was observed in muscle, liver and kidneys of 1-year-old rats [155].

### 1.6. Subcellular Localization of IDE

The subcellular localization of IDE is mainly cytosolic [37,156,157,158], but it has been reported to be present in several other subcellular compartments, including endosomes [58,159,160], peroxisomes [43], mitochondria [107], plasma membrane [161,162,163,164,165], endoplasmic reticulum [166], exosomes [167], the extracellular space [166] and even in human cerebrospinal fluid [168]. If IDE is primarily cytosolic, its role as an insulin protease seems to be called into question. Two main pathways for insulin internalization have been described. At physiological concentrations, insulin is internalized through an IR-mediated process (see reference [1] for a comprehensive review of IDE’s role on insulin uptake and clearance), whereas at higher concentrations a non-receptor mediated uptake internalizes insulin to endosomes [169]. In both pathways, insulin is internalized in endosomes, which begs an important question: How can insulin gain access to the cytosol to be degraded by IDE? Several studies proposed that internalized insulin is probably released to the cytosol by endosomes [170]. The mechanism by which insulin is transported through the membrane of endosomes and enter the cytosol is not well understood, but it has been proposed a two-step process involving acidification of the endosome and unfolding of insulin molecules, which help it pass through the membrane [170]. Modulation of IDE activity may have a significant impact in the accumulation of cytosolic and nuclear insulin or insulin-bound cytoplasmatic proteins [171,172,173,174].

An alternative locus for the interaction between IDE and insulin (and other substrates) is the extracellular space. As mentioned, IDE does not have a signal peptide and is not exported via the classical secretion pathway [81]. Many reports indicate that IDE is secreted in significant quantities from various cell types (e.g., [161,166]), but a recent analysis [82] suggests that IDE release from cultured cells might be non-specific. There is a great need for additional research on this topic, as it is of central significance to the functional role of IDE in regulating the levels of insulin and other IDE substrates.

### 1.7. Transcriptional and Posttranscriptional Regulation of IDE

Although the role of IDE in the regulation of hepatic insulin signaling and glucose homeostasis has been investigated [86,87], the physiological regulation of its expression and activity in hepatocytes remains poorly understood. In human hepatocellular carcinoma HepG2 cells grown in normal glucose medium, exposure to insulin for 24-h did not regulate *Ide* mRNA or protein levels [175]. Likewise, in the presence of high glucose levels, insulin increased expression of *Ide* mRNA, but without changes in levels of IDE [175]. However, insulin increased hepatic IDE activity, but this insulin-mediated effect was abolished in the presence of high glucose levels [175]. The underlying mechanism(s) by which 24-h exposure to insulin regulates IDE activity remains to be deciphered. As mentioned above, human *Ide* mRNA undergoes alternative splicing in exon 15 [153]. Pivovarova and colleagues showed that the relative proportion of the more proteolytically active 15a splice isoform was increased after insulin treatment, independently of glucose levels [175].

Insulin-mediated regulation of IDE has also been investigated in mouse primary hippocampal neurons. Contrary to hepatocytes, exogenous insulin application upregulates IDE protein levels, and this insulin-mediated effect was abolished by inhibition of the insulin-signaling component phosphoinositide 3-kinase (PI3K) [176]. These findings suggest that, within certain cell types, there is a negative feedback mechanism whereby insulin-mediated activation of IR upregulates IDE to prevent chronic activation of the pathway in the presence of high insulin levels.

The effects of glucagon on IDE function in hepatocytes was investigated by Wei and colleagues. In a time-dependent manner, glucagon (100 ng/mL) upregulated IDE protein levels in Hepa 1c1c7 cells. A similar pattern was observed after preincubation of hepatic cells with forskolin (10 µM), an activator of protein kinase A (PKA), suggesting that the glucagon-mediated regulation of IDE proceeds via a cAMP/PKA-dependent pathway [177]. The physiological and pathophysiological relevance of these findings awaits further validation in vivo.

Lin and colleagues demonstrated that Fas-associated protein with death domain (FADD), a classical adaptor in the Fas-FasL signaling pathway, which is phosphorylated in response to members of the tumor necrosis factor receptor family, regulates the expression of IDE at the transcriptional level, without affecting the stability of *Ide* mRNA in HepG2 cells [178]. FADD knockdown in HepG2 cells by siRNA resulted in downregulation of both mRNA and protein levels of IDE without change in IDE mRNA stability. Similar effects on IDE mRNA and protein levels were observed in the liver of mice overexpressing FADD-D (a mimic of constitutively phosphorylated FADD) as well as in primary hepatocytes cultured from these mice, effects that were attributable to reduced stability of IDE protein [178]. Interestingly, in primary hepatocytes from FADD-D mice, nuclear translocation of the transcription factor forkhead box O1 (FoxO1) is enhanced, and the transcriptional activity of the IDE promoter in response to FADD knockdown in HEK293T cells was decreased. Furthermore, the transcriptional activity of the *Ide* promoter was reduced by expressing FoxO1 in HEK293T cells [178]. Altogether, these results point out that FADD phosphorylation may reduce the expression of IDE by promoting the nuclear translocation of FoxO1. The detailed regulatory mechanism by which FADD phosphorylation regulates transcriptional activity of the *Ide* promoter in hepatocytes requires further experimental confirmation. In addition, these findings open an avenue to explore whether the insulin signaling pathway through FoxO1 regulates IDE levels in hepatocytes.

The cannabinoid receptor 1 (CB1) is a seven-transmembrane G protein-coupled receptor present in liver, and its activation by endocannabinoids stimulates lipogenic genes in hepatocytes, leading to increased fatty acid synthesis [179]. Pancellular genetic deletion of CB1 in mice resulted in resistance to diet-induced obesity, and liver-specific deletion in mice fed a HFD showed lower insulin resistance, hyperglycemia and steatosis [179]. In HepG2 cells, the endocannabinoid anandamide, a metabolite of the non-oxidative metabolism of arachidonic acid that is a partial agonist of CB1, causes down-regulation of IDE in a time-dependent manner, in parallel with Ser307 phosphorylation of insulin receptor substrate 1 (IRS1) [180]. Likewise, acute treatment with anandamide or feeding with a HFD reduced hepatic IDE levels, in parallel with insulin resistance in mice. The CB1-mediated regulation of IDE was further corroborated by the finding that hepatic IDE expression is downregulated in mice that overexpress CB1 in hepatocytes (htgCB1 mice) but not in CB1 knockout mice [180]. Of note, htgCB1 mice displayed down-regulation of IDE and its proteolytic activity, but unaltered levels of phosphorylated carcinoembryonic antigen-related cell adhesion molecule 1 (CEACAM1), in parallel with hepatic insulin resistance, lower insulin clearance and moderate hyperinsulinemia [180].

The impact of inflammation mediators on IDE function has also been examined in hepatocytes and pancreatic cells. Interleukin-6 (IL-6) is a pro-inflammatory cytokine that mediates inflammation associated with insulin resistance in the liver and other tissues [181]. In HepG2 cells, exposure to IL-6 increases IDE protein levels [182]. Conversely, in livers of IL-6 knockout mice, which develop glucose intolerance without a change in insulin sensitivity, hepatic insulin clearance is reduced, which is associated with reductions in the levels of IDE mRNA, protein and activity [182]. In addition, IL-6 knockout mice exhibited diminished C-peptide secretion after administration of an intraperitoneal (IP) glucose bolus, and reduced glucose-stimulated insulin secretion in isolated pancreatic islets, leading to lower fasting plasma insulin levels [182]. In the opposite direction of IL-6’s effect on hepatic IDE function, exposure to tumor necrosis factor α (TNFα) decreased IDE mRNA and protein levels in Hepa 1c1c7 cells [177]. Thus, additional work is warranted, but these findings suggest IDE may play a role in the mechanisms linking inflammation to the regulation of pancreatic insulin secretion and hepatic insulin resistance.

In addition to the aforementioned, other modulators can regulate IDE protein and activity levels. Thus, ATP and other nucleoside polyphosphates, as well as polyphosphate alone, induce dose-dependent allosteric inhibition of insulin degradation and, concomitantly, activation of short fluorogenic peptide substrates at physiologically relevant concentrations (1–5 mM) [50,183]. Interestingly, the activating effect is stronger for nucleoside triphosphates than nucleoside di- or monophosphates, and is attenuated in the presence of Mg^2+^ [50,184]. Polyphosphate binding has been shown to occur at a specific region within IDE, known as the polyanion-binding domain [144], and appears to mediate activation of the protease towards short substrates by facilitating the transition from the closed state to the open conformation [49,50]. Camberos and colleagues reported that IDE has ATPase activity [185], but this was not confirmed by other investigators (M. Leissring, unpublished observations) and a molecular mechanism for this functionality is not evident from the crystal structures of IDE [63].

Acidic pH also affects the ability of IDE to bind insulin and alter its degradation by inducing dissociation of the oligomerization state into monomeric units. Since IDE is most active at neutral and basic pH, suggest that cellular acidosis may regulate insulin signaling and degradation [186]. Further studies are necessary to understand the impact of clinically relevant diabetic ketoacidosis, and starvation ketoacidosis stimulated by the combination of low insulin and high glucagon on IDE function.

Nitric oxide (NO) production is known to play an important role in permissive regulation of glucose-stimulated insulin secretion, and hepatic insulin resistance [187,188,189]. In this connection, it is interesting to note that S-nitrosylation of IDE, mediated by S-nitrosoglutathione, a potent physiologically relevant NO donor source in cells, inhibited the proteolytic activity of IDE [96]. Notably, NO donors exert this effect in a non-competitive manner, without affecting insulin binding to IDE or the insulin degradation products it produces [190]. Similarly, Cordes and colleagues showed that NO inhibited the degrading activity of IDE in rat liver homogenates in a dose-dependent manner [191]. Somewhat controversially, Natali and colleagues have postulated that the increased insulin clearance evoked by systemic blockage of endogenous NO synthesis in humans may be accounted for by effects on IDE function in liver [192]; however, the physiological and pathophysiological relevance of this proposed mechanism of IDE regulation on hepatic insulin action and signaling awaits further confirmation.

IDE is vulnerable to oxidative damage in multiple ways. For instance, IDE has been shown to be covalently modified and consequently inactivated by 4-hydroxynonenal (HNE), an oxidative byproduct of lipid metabolism [193,194]. Of note, in the brains of mice, HNE-modified IDE accrues in an age-dependent manner, being particularly abundant in brain regions affecting in Alzheimer’s disease [193]. HNE, H_2_O_2_ and other oxidizing agents act, not only, to inhibit IDE proteolytic activity, but also promote the proteolysis of IDE itself by other proteases [194]. Conversely, dietary vitamin E supplementation, a classical lipophilic antioxidant that protect against free radicals [195], increased IDE mRNA levels in livers of rats, which was associated with improved glucose tolerance and insulin sensitivity [196].

The effects of caloric restriction and exercise on IDE function have been examined in mice, as well. Mice fed a low-protein diet for 14 weeks showed improved glucose tolerance and hepatic insulin sensitivity, in parallel with reduced insulin secretion and clearance, which was associated with ~50% reduction in IDE protein levels in the liver [197]. Likewise, caloric restriction (food restriction of 40%) for 21 days resulted in improved glucose tolerance and insulin sensitivity in rats, which was associated with lower hepatic IDE protein levels [196]. Mice subjected to a single bout of exercise on treadmill for 3-h showed reduced glycemia and insulin sensitivity, in parallel with higher insulin secretion in isolated islets and insulin clearance, which was associated with increased expression of IDE in the liver [198].

Exposure to increasing amounts of free fatty acids (FFA) released from adipose tissue promotes the development of hepatic insulin resistance and impaired glucose-stimulated insulin secretion in pancreatic β-cells [199,200], and some reports suggest that IDE may be either affected by or involved in this phenomenon. Early reports showed that FFA reduced leakage of IDE from isolated rat hepatocytes and inhibited the proteolytic activity of IDE released from adipocytes [201,202]. Svedberg and colleagues investigated the effect of different fatty acids on insulin binding, degradation and action in isolated rat hepatocytes, finding that different fatty acids rapidly decreased insulin binding and degradation in isolated hepatocytes [203]. Furthermore, the effect of FFA was specifically on the rate of insulin receptor internalization and/or recycling [203]. Of note, Hamel and colleagues identified a fatty acid-binding motif within IDE and subsequently examined the effect of FFA and their acyl-coenzyme A thioesters on IDE partially purified from rat livers [204]. They observed that both saturated and unsaturated long-chain FFA, and the corresponding acyl-coenzyme A thioesters, inhibited insulin degradation in a non-competitive manner, but did not inhibit binding of the hormone to IDE [204]. In addition to the effect of FFA on IDE activity, Wei and colleagues investigated the impact of FFA on IDE expression, showing that palmitic acid (300 µM) augmented IDE protein levels in Hepa 1c1c7 cells [177]. These results are not in accordance with effects of FFA on IDE expression in brain, where palmitic acid reduced, while docosahexaenoic increased, IDE protein levels in neuron [205]. Furthermore, palmitic acid attenuated the effect of docosahexaenoic acid in brain [205]. In light of these studies, it is tempting to hypothesize that obese patients are exposed to increasing amounts of saturated FFA (e.g., palmitic acid) released from mesenteric and omental fat via the portal system, which would be predicted to inhibit both proteolytic and non-proteolytic functions of IDE, in turn decreasing insulin clearance, whether directly by reducing IDE levels and/or activity or indirectly via mechanisms involving IR internalization and/or recycling. This mechanism may help explain the insulin resistance and hyperinsulinemia seen in obese patients, but further research is needed to validate this hypothesis.

### 1.8. Pharmacological Modulation of IDE

The proteolytic activity of IDE in vitro is sensitive to non-specific inhibitors such as EDTA (a metal-chelating agent), 1,10-phenanthroline (a Zn^2+^ chelator), p-hydroxymercuribenzoate and iodoacetate (cysteine proteinase inhibitors), N-ethylmaleimide (NEM) and p-chloromercuribenzoate (sulfhydryl-reactive compounds) and bacitracin (cyclic polypeptides from *B. subtilis* that inhibit bacterial growth) [206,207,208]. Importantly, IDE is sensitive to inhibition by sulfhydryl-directed reactions, such as alkylation (NEM) and oxidative inactivation (H_2_O_2_) [145,148,194,209,210]. The multi-functional roles of IDE, and the fact that most existing inhibitors were non-selective, inspired the development of pharmacological inhibitors of IDE. The first potent and selective IDE inhibitor was developed by Leissring and colleagues, who used a rational drug design approach based on analysis of the subsite sequence selectivity of IDE, resulting in Ii1, a highly potent (K_i_ = 2 nM) peptide hydroxamic acid [211]. Another thiol-targeting IDE inhibitor developed by this group, ML345, is of interest because it selectively targets extracellular IDE while sparing cytosolic IDE via the formation of a redox-sensitive disulfide bond [209]. More recently, a potent and commercially available IDE inhibitor, 6bK, was developed by Maianti and colleagues [115], which is highly selective because it targets the exosite rather than the active site within IDE. Further, 6bK is notable because it exhibited multiple antidiabetic properties in vivo [115]. A recent drug-repurposing screen conducted by Leroux and colleagues [212] identified an existing drug, ebselen (EB), a synthetic organoselenium compound with antioxidant and anti-inflammatory properties, as a potent pharmacological inhibitor of IDE (apparent IC_50_ against insulin degradation = 14 nM) [212,213,214,215]. EB was found to inhibit IDE via unusual mechanisms of action. First, EB was shown to be a reversible inhibitor [212], despite the fact that it is known to covalently modify cysteine residues [216], and despite the fact that it was inactive against a cysteine-free form of IDE [212]. While the reversibility of EB action would suggest the compound does not directly modify thiols, an independent study found that a biotinylated form of EB interacts with IDE in a covalent manner [217]. Second, EB was found to shift the quaternary structure of IDE, destabilizing the homodimer and promoting monomer formation [212]. Interestingly, EB has an insulin-mimetic action, reducing hyperglycemia, enhancing glucose uptake in peripheral tissues [218], restoring glucose-stimulated insulin secretion in pancreatic β-cells [219], and improving hepatic insulin signaling and β-cell survival [220], suggesting that EB-mediated IDE inhibition may be involved in the mechanism of action of this compound. Consistent with the idea that IDE inhibition could potentiate insulin signaling, Leissring and colleagues identified peptidic IDE inhibitors via phage display that promote insulin-dependent collagen production in skin fibroblasts and migration in cultured keratinocytes, suggesting these compounds may have therapeutic value for promoting wound healing [221]. Finally, in another interesting application of IDE inhibitors, Demidowich and colleagues proposed the utility of using bacitracin in blood samples to counteract insulin degradation from IDE released during hemolysis, which complicates interpretation of clinical data [222].

Indirect effects of several diabetes drugs, such as thiazolidinediones, on the levels and functionality of IDE have also been investigated. Pioglitazone, an insulin sensitizer that enhances insulin action and decreases hepatic gluconeogenesis in liver [223], increased IDE mRNA and protein levels in a time-dependent manner in the mouse hepatoma cell line Hepa 1c1c7 [177]. In addition, pioglitazone administration resulted in higher IDE mRNA and protein levels in livers of mice fed a HFD concomitant with improved insulin sensitivity and lower circulating glucose and insulin levels [177]. Troglitazone was the first thiazolidinedione to be used in diabetic patients but was subsequently withdrawn for clinical use due to its hepatotoxicity [224]. Troglitazone administration reduced hepatic triglyceride content and decreased de novo lipogenesis, in parallel with higher insulin clearance in rats fed a high-sucrose diet for two weeks. These metabolic improvements were associated with augmented IDE activity, but similar IDE protein levels in the liver [225].

## 2. Studies of Global Manipulation of *Ide* on Insulin and Glucose Tolerance In Vivo

### 2.1. Effects of Pancellular Genetic Deletion of Ide on Insulin and Glucose Tolerance In Vivo

As an initial approach to help elucidate the function of IDE in vivo, several studies were conducted in mice with pancellular deletion of IDE (IDE-KO mice). Farris and colleagues, the first team to investigate diabetes-related endpoints in this mouse model [80], found that 6-month-old IDE-KO mice exhibited elevated fasting plasma glucose and insulin levels along with profound glucose intolerance [80]. Abdul–Hay and colleagues subsequently conducted a longitudinal characterization of IDE-KO mice, conducting glucose and insulin tolerance tests at 2, 4 and 6 months of age [83]. Whereas fasting plasma insulin levels were found to be elevated at all ages in IDE-KO mice, glucose and insulin tolerance transitioned from being modestly improved relative to wildtype controls at 2 months of age to profound glucose and insulin intolerance at 6 months of age [83]. The age-dependent emergence of the diabetic phenotype led Abdul–Hay and colleagues to propose that it arose as a consequence of chronic hyperinsulinemia, reporting that IR levels in muscle, adipose and liver tissue decreased as a function of age [83]. Consistent with the reduction in IR levels, primary adipocytes harvested from 6-month-old IDE-KO mice also showed functional impairments in insulin-stimulated glucose uptake [83]. Finally, IDE-KO mice were also characterized by Steneberg and colleagues [85]. Consistent with previous reports, Abdul–Hay and colleagues found that IDE-KO mice fed a normal diet exhibit pronounced glucose intolerance that increased in severity in an age-dependent manner [85]. Unlike previous studies, however, Steneberg and colleagues conducted a detailed examination of glucose-stimulated insulin secretion, finding that plasma insulin levels were markedly reduced in IDE-KO mice after glucose challenge [85]. Subsequent ex vivo and in vitro analyses yielded confirmatory evidence that insulin secretion was indeed impaired by deletion of *Ide* [85], as discussed in greater detail in Section 3. Collectively, these contradictory studies raise far more questions than they answer, suggesting that analysis of mice with global deletion of IDE might not be the most helpful approach to elucidating the precise role(s) of IDE in regulating insulin and glucose homeostasis.

Given the large number of substrates processed by IDE, its widespread expression in various tissues and subcellular organelles, as well as the various proteolytic and non-proteolytic functions of IDE and its homologs and paralogs, pancellular deletion of IDE might be expected to result in a large number of diverse phenotypes. It is therefore of some note that IDE-KO mice have so far been shown to exhibit very few phenotypic differences unrelated to metabolism. Apart from elevations in Aβ levels in brain already discussed, IDE-KO mice were recently shown to exhibit reduced sperm quality associated with substantial decreases in testes weight and seminiferous tubule diameter [226]. While interesting, these phenotypic changes may be secondary to the metabolic disturbances in IDE-KO mice, since reduced fertility and impaired sperm quality is a known consequence of diabetes [227]. Given that testes express transcripts for both insulin [228] and *Ide* (particularly the 15b splice isoform) [153], further research on this topic is warranted.

### 2.2. Effects of Pharmacological Inhibition of IDE on Insulin and Glucose Tolerance In Vivo

Based on the ability of IDE to avidly degrade insulin, investigators have, for more than a half century, postulated that pharmacological inhibition of this protease could augment insulin action by prolonging the half-life of circulating insulin, thereby improving glucose homeostasis in diabetic patients [229]. Stimulated in part by this rationale, several pharmacological inhibitors of IDE have been developed [38,115,211,230,231,232,233,234]. IDE inhibitors are highly desirable from an experimental perspective, as well. For instance, unlike gene knockout approaches, pharmacological inhibition of IDE allows the effects of acute inactivation of IDE to be investigated, obviating compensatory changes that might accrue over longer time frames in knockout mice, while also theoretically permitting the experimental separation of proteolytic and non-proteolytic functions of IDE. Although several highly potent and selective compounds have been generated, these compounds have exhibited mixed effects on glucose tolerance and circulating insulin levels when tested in vivo. In line with the long-predicted outcome, Maianti and colleagues found that the IDE inhibitor 6bK improved oral glucose tolerance and insulin tolerance in normal and diabetic mice, with no effect evident in IDE-KO mice [115]. Intriguingly, however, 6bK dramatically worsened IP glucose tolerance 1 h after administration [115], a distinction that was attributed to the “incretin effect” and the associated involvement of different hormones [235]. Consistent with the latter idea, 6bK produced increases in insulin, amylin and glucagon, albeit with different temporal profiles [115]. Another mouse study involved the use of the dual-exosite targeting IDE inhibitor, NTE-1, developed by investigators at Eli Lilly [233]. As was true for 6bK, NTE-1 administration also improved the glucose excursion in oral glucose tolerance tests in diet-induced obese mice [233]. However, insulin tolerance was unchanged, and no increases in insulin levels were observed; moreover, euglycemic clamping studies revealed no changes in insulin responsiveness [233]. Interestingly, the researchers found no effect on exogenous insulin clearance in HEK cells treated with the related inhibitor, NTE-2, whether or not these cells expressed the IR; similarly, no effect of IDE overexpression or downregulation was observed in the latter cells [233]. Notably, however, NTE-1 treatment did lead to increases in circulating levels of amylin in vivo [233]. Yet another IDE inhibitor, BDM44768, developed by Deprez–Poulain and colleagues, was also evaluated in mice [234]. Mice treated with BDM44768 exhibited modestly improved insulin tolerance along with modestly increased plasma insulin levels after IP insulin administration [234]. Strikingly, however, BDM44768 administration resulted in a significant worsening of oral and IP glucose tolerance in both wildtype B6 mice and non-obese diabetic mice but not B6 or NOD mice lacking IDE, suggesting the effect was indeed dependent on IDE [234]. Of note, plasma insulin levels were increased after glucose challenge in NOD but not wildtype mice treated with BDM44768 [234]. Moreover, hepatic gluconeogenesis was not altered by BDM44768 as assessed by pyruvate tolerance testing [234]. Significantly, BDM44768 increased in a dose-dependent manner the amount of insulin secreted by isolated islets obtained from wildtype, but not IDE-null, mice [234]. The ratio of insulin secreted in response to high versus low levels of glucose was not altered, however, leading the investigators to conclude that BDM44768 does not influence the responsiveness of β-cells to glucose [234].

Taken together, these in vivo pharmacological studies paint a complicated picture, with some supporting a role for IDE in regulating insulin levels and others confuting this idea. Similarly, some IDE inhibitors exhibited antidiabetic properties in oral glucose tolerance tests, but the opposite was observed for other inhibitors or in other tests of glucose and insulin tolerance. It seems evident that the picture is so complicated in large part because IDE degrades multiple hormones with overlapping and/or contradictory effects on blood glucose. In light of this, it is relevant to note that Maianti and colleagues recently developed an insulin-selective IDE inhibitor [236]. Future studies with this inhibitor hold great promise for disentangling the role of IDE in insulin homeostasis independent of its effects on amylin, glucagon and other substrates.

## 3. Role of IDE in Pancreatic β-Cells

### 3.1. IDE Protein Expression in Pancreatic β-Cells

Despite being a widely studied cell type in T2DM, the pancreatic β-cell and precisely how it malfunctions in disease, remains incompletely understood. Several pathogenic mechanisms have been proposed, including cell death [237,238], de-differentiation [239,240], trans-differentiation [241,242] and loss of cell identity [243,244] have been proposed. Understanding the molecular mechanisms underlying these processes will help to develop new strategies to recover β-cell function in T2DM.

With respect to IDE, several genome-wide association studies conducted on large populations have independently identified genetic variations in and around the *Hhex*/*Ide* locus that are associated with T2DM incidence, decreased insulin secretion, and differential β-cell glucose sensitivity in response to an oral glucose challenge [141,198,245,246,247]. However, until recently the biology of IDE in the β-cell remained largely unknown. For the first time, in 2018, Fernández–Díaz and colleagues investigated the expression pattern of IDE in pancreatic islet cells [157]. We showed that IDE is differentially expressed in different pancreatic islet cells, being expressed to substantially higher levels in pancreatic α-cells relative to β-cells and all other islet cell types. This finding suggests that it may be relevant to investigate the role of this protease in glucagon-producing cells in future research [157].

There is some evidence suggesting a potential role for IDE in T2DM-associated β-cell dysfunction, although the literature on certain topics is contradictory [85,248,249,250,251]. The abundance of contradictory findings likely reflects the multifactorial role that IDE plays in different tissues and cell types, highlighting the need for cell-specific manipulations to deepen our understanding of IDE’s various functions. Regarding pancreatic β-cells, by western blotting, Steneberg and colleagues showed that IDE protein levels are diminished by 40% in whole islets from T2DM donors compared to controls [85], a finding that was later corroborated by Fernández–Díaz and colleagues via immunostaining [157]. Furthermore, IDE expression levels were modulated in T2DM patients, depending on the treatment they received. IDE appeared decreased in diabetics treated with oral hypoglycemic agents, which was in line with Steneberg and colleagues’ observations in whole pancreatic islets from diabetic donors (whose treatment was unspecified) [85]. On the other hand, insulin-treated patients, showed increased IDE protein levels in pancreatic β-cells relative to patients treated with oral hypoglycemic agents, pointing to an upregulation of IDE under high insulin conditions [157]. This hypothesis was confirmed in multiple experimental paradigms, including preclinical murine models with hyperinsulinemia (*db*/*db* mice and high fat-fed mice) as well as in in vitro experiments using insulin administration to INS1E cells, as well as to rodent and human pancreatic islets. These consistent findings strongly support the notion that IDE levels in pancreatic β-cells increase in response to insulin exposure [157], in agreement with observations in other cell types, including hepatocytes [175] and primary hippocampal neurons [176].

Why precisely IDE levels increase in response to elevated insulin is unknown, but we can speculate that this may occur as a counter-regulatory adaptative mechanism for clearing excessive insulin and thereby restoring homeostasis. This idea is supported by the observation that insulin that is not cleared by liver and kidney is ultimately removed by other insulin-sensitive cells [37]. Taken together, these studies reveal that IDE expression in pancreatic β-cells is remarkably plastic, varying in response to different metabolic and hormonal milieus to preserve β-cell function.

### 3.2. Effects of Genetic Deletion of Ide on Insulin Secretion In Vitro

The main function of a pancreatic β-cell is the production and secretion of insulin in response to increased circulating glucose levels. To test how IDE is involved in this process, Fernandez–Diaz and colleagues treated both rat and human islets with the IDE inhibitor NTE-2 [123,233]. The result was clear: Transient inhibition of IDE led to an abolition of glucose-stimulated insulin secretion (GSIS), thus demonstrating the crucial role of IDE in β-cell function. This pattern of results was also obtained in INS1E cells, an immortalized β-cell line, following transient downregulation of *Ide* by RNA interference. Of note, these results are consistent with the impaired release of insulin associated with genetic variations around the *Hhex*/*Ide* chromosomal locus in humans [247,252] and with Steneberg and colleagues’ findings, who reported deficient insulin secretion from islets isolated from IDE-KO mice [85].

Confirming these observations, shRNA-mediated silencing of *Ide* in immortalized β-cells (INS1E-shRNA-IDE cells) resulted in decreased insulin secretion in response to glucose [123]. These investigators observed increased intracellular insulin content in INS1E-shRNA-IDE cells and electron microscopy images revealed elevated numbers of insulin granules in the cytoplasm, pointing to a delayed movement of the granules through the cytoplasm during insulin secretion. This mechanism would be in agreement with previous observations showing that decreased insulin release caused by IDE ablation is related to impaired polymerization of α-synuclein, a protein involved in the reorganization of cellular microtubules [85]. One of the limiting steps during insulin secretion is the correct organization of microtubules: These allow insulin granules to travel through cytoplasm to reach the cell membrane and release insulin to the medium in response to increased extracellular glucose levels [253]. Thus, the role of IDE in regulating glucose-stimulated insulin secretion in β-cells appears to be related to this non-proteolytic function rather that to its degradative capacity.

On the other hand, because loss of IDE function in β-cells resulted in intracellular accumulation of insulin, it is plausible to propose a proteolytic role for IDE on regulating insulin levels in mature-beta granules. To maintain insulin stores at optimal levels, mature-insulin granules of the pancreatic β-cells gain access to a lysosomal compartment by crinophagy and/or autophagy where the secretory granule content is degraded [254,255,256]. If IDE could gain access to mature granules at neutral and/or basic pH conditions, the proteolytic activity of IDE might conceivably regulate the insulin content in mature secretory granules. Whether this speculative mechanism is operative in β-cells remains unclear, however, and more studies are warranted.

### 3.3. Impaired Insulin Secretion and β-Cell Immaturity in the B-IDE-KO Mouse

As discussed, to assess whether IDE might be a valid therapeutic target for T2DM treatment, many authors have investigated the effects of pancellular *Ide* deletion in mice models [38,80,83,85,115]. The results obtained are somewhat contradictory, but most reports indicate that genetic deletion of *Ide* results in deleterious metabolic effects, such as impaired glucose homeostasis. However, it is difficult to draw conclusions about IDE’s specific function in β-cells in IDE-KO mice due to potential secondary or compensatory effects of loss of IDE function occurring in other tissues, such as liver. To overcome this problem, and thereby help elucidate the specific role of IDE in β-cells, Fernandez–Diaz and colleagues generated β-cell-specific *Ide* knock-out mice (B-IDE-KO) [123].

In contrast to the previously reported phenotype of IDE-KO mice, B-IDE-KO mice did not fully recapitulate an impairment in glucose homeostasis. Surprisingly, B-IDE-KO mice showed no changes in fasting or non-fasting plasma glucose levels. Glucose homeostasis measured by IP glucose tolerance tests was normal, but after 6 months of age, mice showed glucose levels significantly increased 15 min after glucose challenge, pointing to modest glucose intolerance. Unexpectedly, plasma C-peptide levels were increased in B-IDE-KO as compared to wildtype mice, indicating perturbed regulation of insulin secretion. This observation could have two explanations: Either β-cell mass was increased by *Ide* deletion or β-cells were hypersecreting insulin. Further experiments showed that β-cell area and islet number were normal, and the elevation in C-peptide levels was instead found to be attributable to constitutive insulin secretion. Specifically, B-IDE-KO isolated islets showed impairments in GSIS in the presence of high glucose, but continued to secrete insulin even in the presence of low glucose levels. This impairment in GSIS agrees with the findings of Steneberg and colleagues in islets isolated from IDE-KO mice [85] and with previous in vitro studies using NTE-2 inhibitor and INS1E-shRNA-IDE in β-cells [123]. Interestingly, B-IDE-KO mice exhibited decreased levels of the glucose transporter GLUT2 and increased levels of GLUT1 in the plasma membrane of β-cells. These findings are in agreement with the constitutive secretion seen in B-IDE-KO islets. On the one hand, GLUT1 is operative at low glucose concentrations (1–3 mM) [257], so the elevated levels of GLUT1 would promote insulin secretion at low glucose concentrations. On the other hand, GLUT2 is maximally functional at high glucose concentrations (15–20 mM), so the decreased levels in B-IDE-KO β-cells would account for the impaired response to high glucose [257,258,259,260,261].

The aforementioned perturbations to GLUT1 and GLUT2 levels also suggest a plausible explanation for the elevated fasting plasma insulin levels reported in IDE-KO mice studies [80,83], which was previously attributed to decreased hepatic catabolism of insulin. Because liver-specific deletion of IDE (L-IDE-KO mouse) showed normal plasma insulin levels [86], the hyperinsulinemia seen in the IDE-KO might be due to alterations in the GLUT1/GLUT2 ratio in pancreatic β-cells. In this context, we note that insulin in plasma from wildtype mice would be vulnerable to degradation by any IDE released by hemolysis, which would be absent in plasma from IDE-KO mice, which could conceivably account for the apparent hyperinsulinemia in IDE-KO mice [222]. Ideally, quantification of plasma C-peptide and insulin levels in IDE-KO mice would help to clarify if the hyperinsulinemia seen in IDE-KO mice is related to pancreatic β-cell function or hepatic insulin clearance.

Livers isolated from B-IDE-KO also display increased expression of the hepatic gluconeogenic genes phosphoenolpyruvate carboxykinase (*Pck1*) and glucose-6-phosphatase (*G6pc*), suggesting hepatic insulin resistance, possibly caused by increased flux of insulin through portal vein to the liver. Hepatic insulin resistance could also be the cause of the mild glucose intolerance observed in B-IDE-KO mice. Taken together, these results implicate IDE as a protein that mediates crosstalk between liver and pancreas to maintain insulin and glucose homeostasis.

Constitutive insulin secretion has been reported as a hallmark of β-cell dysfunction and immaturity [262,263,264,265]. In embryonic and neonatal stages, β-cells secrete insulin in a constitutively manner [262,263,264,265]. Extending this line of thinking, Fernandez–Diaz and colleagues speculated that the expression of GLUT1 is increased in embryonic tissues to help meet the demand of energy necessary for the rapid growth of fetal cells [260]. In addition, it is noteworthy that GLUT2 has been reported to be decreased in the plasma membrane of immature and dysfunctional β-cells [264,265,266,267]. Together, these findings are consistent with the conclusion that genetic deletion of *Ide* disrupts the maturation of β-cells, leaving them in a premature metabolic state. 

Islets isolated from B-IDE-KO mice also showed an increase in the secretion of proinsulin in parallel with decreased *Pcsk1/3* expression levels, leading to the secretion of immature insulin granules into the extracellular space, which is another characteristic of immature β-cells [264]. In addition, B-IDE-KO islets showed decreased expression of key genes such as *Ins2* and *Ucn3*, which are necessary for the correct maturation and function of β-cells [262].

In summary, the B-IDE-KO mouse model uncovered an unexpected new IDE function in regulating pancreatic β-cell maturation. These results are consistent with the finding that, during rat development, IDE is differentially expressed in different tissues and in an age-dependent manner [2,75].

## 4. Role of IDE in Liver

### 4.1. Metabolic Phenotype of the L-IDE-KO Mouse

Historically, the study of IDE, and speculation about its proposed function, has been focused on the liver, but most studies were limited to the analysis of post-mortem tissue or cultured hepatocytes. The study of IDE-KO mice offered new possibilities for elucidating IDE’s function in liver; however, as discussed above, the various studies of these animals have yielded conflicting results. Moreover, by virtue of the sheer complexity of the underlying endocrinology, and the many potential proteolytic and nonproteolytic functions of IDE, the study of animals with pancellular deletion of IDE is of limited value—and may in fact yield confounding results. To investigate the role of IDE in liver in a more focused manner, our group generated a mouse line with selective ablation of *Ide* exclusively in hepatocytes, known as the L-IDE-KO model [86].

L-IDE-KO mice exhibit higher fasting and non-fasting glucose levels, glucose intolerance, and insulin resistance, despite normal plasma insulin levels [86]. Of note, plasma levels of other IDE substrates—including glucagon, amylin and Aβ—remained unchanged in L-IDE-KO mice [86]. Contrary to historical predictions about IDE’s function in liver, clearance of exogenously administered insulin was unaltered by hepatic ablation of *Ide*. Moreover, assessment of β-cell function (insulin and C-peptide plasma levels), and histomorphological analyses of pancreas (β-cell mass, β-cell area, number of islets, and mean islets size) revealed that β-cell function and mass in L-IDE-KO mice were similar to wildtype controls [86]. These findings indicate that hepatic *Ide* ablation causes insulin resistance independently of any effect on circulating insulin levels, suggesting that the hyperinsulinemia observed in IDE-KO mice emerged as a secondary compensatory response to systemic insulin resistance.

Another highly intriguing observation made in L-IDE-KO mice involved carcinoembryonic antigen-related cell adhesion molecule 1, a substrate of the IR in liver, that upregulates receptor-mediated insulin endocytosis and degradation in a phosphorylation-dependent manner [268]. As expected, administration of insulin to wildtype mice resulted in robust phosphorylation of CEACAM1 [86]. In marked contrast, however, insulin administration to L-IDE-KO mice resulted in no detectable phosphorylation of CEACAM1, despite unchanged CEACAM1 protein levels [86]. The potential consequences of these observations for insulin resistance are considered in greater detail, below.

To delve more deeply into IDE’s role in the pathogenesis of hepatic insulin action, Merino and colleagues fed L-IDE-KO mice a Western HFD (35% carbohydrates and 45% fat) [87]. As was true for animals fed a regular diet [86], L-IDE-KO fed a HFD mice exhibited insulin resistance and glucose intolerance [87]. Unlike the regular diet [86], however, feeding a HFD to L-IDE-KO mice triggered elevated fasting and non-fasting plasma insulin levels, but normal glucagon levels, relative to control mice fed a HFD [87]. The hyperinsulinemia in HFD-fed L-IDE-KO mice could theoretically be attributable to reduced hepatic insulin extraction and/or enhanced β-cell function and mass. Similar to the case with a regular diet [86], however, hepatic insulin clearance and β-cell mass remained unchanged in L-IDE-KO mice fed a HFD [87]. On the other hand, β-cell function was improved, most likely as a compensatory response to insulin resistance triggered by loss of hepatic IDE function [87].

From a mechanistic point of view, the hepatic insulin resistance present in L-IDE-KO mice appears to be related to diminished insulin action in the liver under regular and HFD feeding [86,87]. Hepatic ablation of IDE causes a reduction in IR protein levels and insulin-mediated phosphorylation of IR, leading to lower AKT (protein kinase B) activation and aberrant nuclear distribution of FoxO1, which in turn enhances expression of gluconeogenic genes (*G6p6* and *Pck1*). Interestingly, hepatic *Ide* ablation altered IR levels post-translationally, as IR mRNA levels were unaffected, and did not reduce protein or mRNA levels of the insulin-like growth factor-1 receptor, which exhibits 70% homology to IR and shares some insulin-responsive signaling pathways [86,87].

The effects on IR regulation seen in L-IDE-KO mice fed a regular diet (which exhibit normoinsulinemia) resemble those seen in pancellular IDE-KO mice (which exhibit hyperinsulinemia). In pathophysiological conditions, such as obesity and T2DM, it is well-documented that cellular IR levels decrease [269,270,271,272] and, moreover, that hyperinsulinemia is associated with accelerated IR degradation [273]. The rate of IR degradation, in particular, is an important factor for controlling the receptor levels in hepatocytes and, hence, their sensitivity to insulin. Because insulin binding to its receptor initiates insulin action, it is apparent that a decrease in IR levels could lead to insulin resistance, but this relationship is not always so clear due to the “spare receptor” concept [274], which is based on the observation that a maximal insulin effect is achieved at an insulin concentration that occupies less than the total number of cellular receptors (10% in adipocytes [274] and 20% in skeletal muscle [275]). Therefore, at any given point in time, the cellular response to increasing insulin levels increases linearly with receptor occupancy, and the maximal biological response occurs when a particular number of receptors are occupied. Beyond this point, increased occupancy of receptors by insulin does not lead to further increases in biological action of the hormone, because events downstream of IR binding become the rate-limiting steps. Thus, the predicted consequence of a progressive loss of IRs on insulin action would be no change in maximal insulin response as long as enough receptors are present, albeit with more insulin required to achieve the same response. However, if the progressive loss of IR reaches a critical threshold (<10–20% of total), the dose-response to insulin and maximal insulin response will diminish drastically. In L-IDE-KO mice fed a regular diet, IR levels are reduced by ~30%, hypothetically leaving sufficient receptors (~70%) above the critical threshold for maximal insulin response [87]. However, because there is no increase in plasma insulin levels, maximal response to the hormone is not achieved, resulting in insulin resistance. On the other hand, L-IDE-KO mice fed a HFD also show a ~30% reduction in IR levels, but also exhibit hyperinsulinemia, which would theoretically permit maximal insulin action. In this case, however, insulin action is instead blocked downstream of the IR, as evidenced by 75% reduction in intracellular AKT levels response to insulin, leading to insulin resistance by this alternative mechanism [87].

Considering that IDE exhibits numerous non-proteolytic functions, such as regulating cytoskeletal components, protein turnover, and/or subcellular localization of proteins [119,125,129,130,134,136], it is tempting to hypothesize that IDE may regulate intracellular trafficking of the IR independently of its protease activity. We recently proposed a coordinated model of IR trafficking and insulin metabolism by CEACAM1- and IDE-dependent pathways [1]. In this model, CEACAM1 promotes the targeting of IR for degradation in response to insulin, and the main effect of IDE would be on IR recycling to the plasma membrane, an important step in insulin retro-endocytosis. Our analysis of L-IDE-KO mice has generated several important findings in support of this hypothesis. As mentioned, hepatic ablation of IDE reduces both the levels and phosphorylation of the IR [86,87]; moreover, depletion of IDE completely abrogated insulin-induced phosphorylation of CEACAM1 on the membrane of hepatocytes [86]. Finally, as considered in greater detail below, Merino and colleagues found that hepatic overexpression of IDE leads to co-immunoprecipitation with the IR in response to insulin [87].

While this model is compelling, we cannot exclude the possibility that, in addition to the non-proteolytical action of IDE, its proteolytic function may regulate insulin action and/or the fraction of bound insulin available for internalization in hepatocytes. The first and the rate-limiting step for insulin action and internalization is its binding to the IR at the plasma membrane. From this point, two cellular processes for insulin metabolism have been proposed [276]. On one hand, insulin degradation has been proposed to occur at the cellular membrane, which does not involve internalization of insulin bound to its receptor. This pathway is bacitracin-sensitive and may therefore involve IDE and/or the glutathione-insulin transhydrogenase, accounting for half of the cellular insulin degraded [277,278,279]. A second pathway requires internalization of the complex IR-insulin into coated pits and the formation of cytoplasmic endosomes. A fraction of the internalized insulin is recycled to the membrane and released intact to the extracellular space (the retroendocytotic pathway), whereas the remained insulin is degraded in endosomes (the degradative pathway) [280]. Acidification of the interior of endocytotic vesicles due to proton pumps facilitates dissociation of the insulin bound to its receptor and degradation of the hormone, most likely by the aspartyl protease cathepsin D [59]. Because IDE activity is pH-dependent, being most active at pH 8.5 [281], it has been suggested that IDE can degrade the B-chain of receptor-bound insulin in light endosomes (early endosomes) prior to endosomal acidification (late endosomes) [57,160,282,283]. In any case, the extent to which proteolytic activity of IDE participates in the regulation of insulin action in hepatocytes, if at all, remains to be determined.

### 4.2. Metabolic Phenotype of Hepatic IDE Gain of Function in Mice

Merino and colleagues also examined the consequence of a gain-of-function manipulation to hepatic IDE in vivo [87]. To that end, an adenovirus IDE expression construct was administered to mice, resulting in ~4-fold increase in liver IDE levels. In mice fed a HFD, hepatic IDE overexpression improved glucose tolerance and insulin sensitivity independently of changes in body weight or food intake [87]. Moreover, plasma insulin and C-peptides levels, but not glucagon, were reduced by hepatic IDE overexpression [87]. Although the reduction in plasma insulin levels might theoretically be explained by increased hepatic insulin clearance by IDE, insulin clearance was found to be unaltered by hepatic IDE, suggesting instead that as a consequence of improved insulin sensitivity the pancreas reduced insulin production and secretion to meet the demand for the hormone in peripheral tissues. This study is the first proof-of-principle demonstration that augmenting hepatic IDE function in liver can partially revert insulin resistance and glucose intolerance in a preclinical mouse model of obesity and diabetes. The opposing effects of loss versus gain of IDE function on insulin levels and glucose tolerance are consistent with a role for IDE in promoting insulin sensitivity in liver of diet-induced obese mice. In a similar way, Leissring and colleagues demonstrated that transgenic upregulation of IDE in neurons, significantly reduces brain Aβ levels, reduced amyloid plaque formation, and rescued the premature lethality present in amyloid precursor protein transgenic mice [284]. Because reduced IDE function has been implicated in the pathogenesis of both T2DM and Alzheimer disease [142], these studies lend support the notion that pharmacological upregulation of IDE might represent viable therapeutic strategies for the treatment of both diseases.

Interestingly, both gain and loss of IDE function in mice fed a HFD resulted in reductions in total IR protein levels [87]. We hypothesized that this occurs because, on the one hand, depleting IDE reduces IR recycling, while on the other, IDE overexpression speeds up IR turnover. In support of this notion, Li and colleagues showed that insulin increased colocalization and co-immunoprecipitation of IDE and SNX5 in plasma membrane of kidney cells (an important organ for systemic circulation insulin clearance and insulin-mediated gluconeogenesis), whereas loss of SNX5 function led to reduced IDE protein and activity, in parallel with decreased expression of the IR and downstream insulin signaling [134]. Further studies are necessary to more fully delineate the molecular mechanisms by which IDE regulates hepatic IR protein levels as well as the physiological and pathophysiological relevance.

In addition to regulation of IR levels, manipulation of hepatic IDE also alters glucose transporters levels. Thus, loss of IDE function in HFD fed mice resulted in a two-fold increase in GLUT2 protein levels, with a reciprocal two-fold reduction of GLUT2 protein levels in mice overexpressing IDE. In addition, hepatic IDE gain-of-function resulted in a two-fold increase in GLUT1 protein levels, therefore altering the hepatic GLUT1/GLUT2 ratio [87]. Taken together, these findings lend support to the notion that IDE forms complexes with membrane proteins to regulate the intracellular trafficking of the IR independently of its proteolytic function.

### 4.3. Novel Insights into the Etiology and Pathophysiology of Hepatic Insulin Resistance: Lessons from Knockout Mouse Models

Over the past several decades, the study of proteins involved in the regulation of the insulin signaling pathway, and liver knockout mouse models in particular, have generated novel insights into the etiology and pathophysiology of hepatic insulin resistance (Table 3). For most of them, a defect in the insulin signaling pathway translated to hepatic insulin resistance and hyperinsulinemia, with the exception of hepatic ablation of FoxO1, which exhibits normoinsulinemia and heightened insulin sensitivity [285], and hepatic Akt2, which showed indistinguishable insulin sensitivity as compared to control mice [286,287].

A signature of the L-IDE-KO mouse model is the presence of hepatic insulin resistance without associated hyperinsulinemia, leading to augmented blood glucose excursions under normal conditions. Conversely, in the setting of obesity induced by a HFD, loss of IDE function exacerbates hyperinsulinemia and worsens glucose intolerance in wildtype mice fed a HFD [86,87]. Applied to the etiology of T2DM, these observations suggest that loss of IDE function represents an early step of the development of T2DM in healthy individuals, triggering impaired glucose tolerance and/or impaired fasting glycemia, before beginning the compensatory hyperinsulinemic phase. On the other hand, for overweight or obese patients with impaired glucose intolerance or fasting glycemia, loss of IDE function would accelerate the compensatory hyperinsulinemia and eventually facilitate the onset of T2DM. More studies are necessary to demonstrate the cause-effect relationship between hepatic IDE function and the onset of T2DM in lean and overweight/obese patients.

Because L-IDE-KO mice were generated using the Cre/loxP system harboring a null allele in their germline, the metabolic phenotype of these mice is related to pre- or postnatal *Ide* deficiency, and metabolic adaptations may arise across lifespan. Thus, the development of an inducible L-IDE-KO mouse line would be valuable, as it would permit the analysis of disruption to IDE function occurring in adult mice, in the absence of disruptions to IDE function during development. An inducible L-IDE-KO model of study would also help elucidate the impact of IDE function on insulin sensitivity and glucose homeostasis before and after the onset of diabetes in mouse models of T2DM such as the *db*/*db*.

By way of conclusion, to our best knowledge, the L-IDE-KO is one of the few knockout mice models of hepatic insulin resistance in which insulin clearance has been assessed in normal and HFD feeding. So far, the liver CEACAM1 knockout mice provide an in vivo proof of the key role of impaired hepatic insulin clearance and hyperinsulinemia in the pathogenesis of secondary hepatic insulin resistance. Considering the importance of insulin clearance for the regulation of circulating insulin levels, it would be of interest to investigate how IDE levels and activity are impacted in mouse models with liver-specific deletion of, for example, IR, IRS1/2, PI3K and rictor, which could help to elucidate the mechanistic basis for the hyperinsulinemia occurring in these models (Table 3), and its impact on IDE levels and activity.

## 5. Concluding Remarks

**Figure 3:**
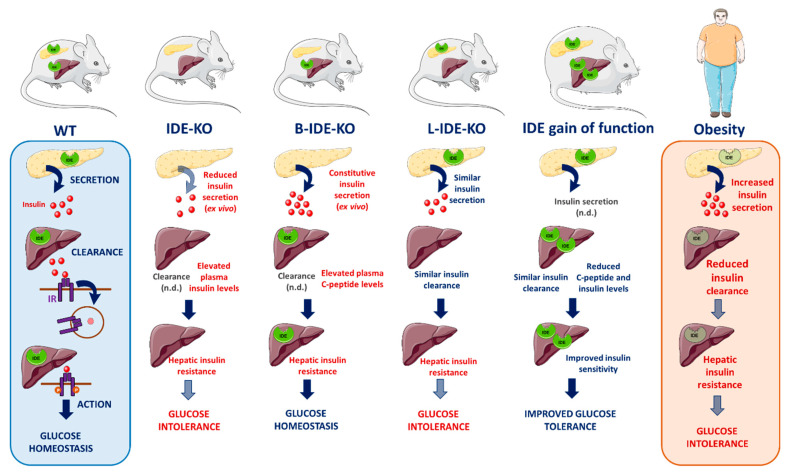
IDE mouse models for the study of insulin proteostasis and insulin sensitivity. In wildtype mice (WT), glucose homeostasis is regulated by insulin secretion out of the pancreas and clearance in the liver. In the fed state, high glucose levels stimulate pancreatic β-cells insulin secretion into portal vein, which is extracted by an insulin receptor-mediated process in hepatic cells. In parallel, insulin promotes glucose utilization and suppresses glucose production in hepatocytes. Pancellular genetic deletion of *Ide* (IDE-KO mice) causes hyperinsulinemia, hepatic insulin resistance, and glucose intolerance, but isolated islets exhibit reduced insulin secretion. Genetic deletion of *Ide* in pancreatic β-cells (B-IDE-KO mice) is associated with elevated plasma C-peptide levels, most likely due to constitutive insulin secretion, leading to hepatic insulin resistance, albeit normal glucose tolerance. Genetic deletion of *Ide* in hepatocytes (L-IDE-KO mice) results in hepatic insulin resistance and glucose intolerance, without altering insulin secretion and clearance. Conversely, IDE overexpression in liver improves hepatic insulin resistance and glucose intolerance, without altering insulin clearance in diet-induced obese mice. Finally, IDE levels are reduced in pancreatic β-cells and the liver of obese patients, which associates with hyperinsulinemia, reduced hepatic insulin clearance, hepatic insulin resistance and glucose intolerance. Each one of the IDE mouse models display hallmarks of the metabolic alterations seen in the setting of obesity. This figure was created using Servier Medical Art (available at https://smart.servier.com/). n.d., not determined.

More than 70 years ago, IDE was first identified as the protease that predominantly degrades insulin. This finding immediately suggested a major role for this protein in the regulation of insulin homeostasis via hepatic insulin clearance. As revealed by this comprehensive review of numerous aspects of IDE biology, with an emphasis on its role in the regulation of insulin secretion and insulin resistance, the biology of IDE has proven to be considerably more complex. Knockout mouse models have demonstrated that the physiological processes regulated by IDE are much broader than expected and, in particular, strongly implicate non-proteolytic functions of this enzyme. These mouse models reveal that neither loss nor gain of hepatic IDE function affected plasma insulin levels or insulin clearance, with the important caveat that hepatic insulin clearance in L-IDE-KO mice has not yet been evaluated by hyperinsulinemic-euglycemic clamping. Furthermore, in B-IDE-KO mice, loss of IDE function alters the expression of key genes necessary for correct maturation of β-cells, leading to the secretion of immature granules and constitutive insulin secretion independent of glucose levels. These findings underscore the importance of tissue-specific knockout mouse models for unravelling the IDE’s roles in regulating insulin metabolism and action (Figure 3).

The L-IDE-KO and B-IDE-KO mouse models have also helped highlight the notion that IDE may participate in the crosstalk between the liver and β-cells. Thus, hepatic loss of IDE function in the setting of diet-induced obesity enhanced β-cells function, leading to increased insulin secretion and hyperinsulinemia to help counteract hepatic insulin resistance. On the other hand, loss of IDE function in β-cells increased expression of hepatic gluconeogenic genes as a result of hepatic insulin resistance, most likely due to increased flux of insulin through the portal vein to the liver. Finally, hepatic IDE overexpression in diet-induced obese mice improves insulin sensitivity and decreases circulating insulin levels. Further studies will be needed to more rigorously assess this idea, but it seems evident that altering IDE function in liver or pancreas reciprocally modifies insulin action and secretion in these tissues.

The less prominent but equally important aspects of IDE function that reside beyond of its proteolytic effect on insulin have been noted for decades, but remained poorly clarified. Numerous studies have found that IDE interacts directly or indirectly with proteins not related to insulin metabolism, such as transcriptional factors, cell receptors, and cytoskeleton. Here, we present evidence that IDE regulates, in both liver and pancreas, glucose transporters (GLUT1 and GLUT2) and hepatic IR levels, suggesting a non-proteolytic role of IDE in the regulation of intracellular trafficking of proteins involved in the regulation of insulin sensitivity and glucose tolerance. The precise molecular and biochemical mechanism(s) by which IDE regulates intracellular trafficking in response to insulin, and its relevance for insulin sensitivity, remains to be deciphered, but is a very attractive idea that warrants additional research.

Research during the coming years may provide answers as to several outstanding questions. Is pharmacological inhibition of IDE a viable approach to the treatment of T2DM? Does the proteolytic activity of IDE play any role in hepatic insulin clearance? How do perturbations to IDE alter insulin sensitivity? Does IDE physically interact with different components of intracellular insulin signaling pathways? How do perturbations of IDE affect pancreatic function and islet maturity? The answers to these and other questions will facilitate our understanding of the etiology and molecular pathogenesis of T2DM and, hopefully, might stimulate the development of novel therapeutic approaches to treating, preventing or potentially reversing this increasingly common disease.

## Figures and Tables

**Table 1:** Orthologs of insulin-degrading enzyme (IDE).

UniProtKB Entry	Organism	Gene Name	Protein Name	Protein Length	E-Value *	Identity (%) *	Positives (%) *
Q5UPX9 (YL233_MIMIV)	*Acanthamoeba polyphaga mimivirus (APMV)*	*MIMI_L233*	Putative zinc protease L233	440	1.3 × 10^−^^5^	20.9	40.4
P31828 (PQQL_ECOLI)	*Escherichia coli* *(strain K12)*	*pqqL*	Probable zinc protease PqqL	931	2.4 × 10^−^^9^	24.6	41.5
P05458 (PTRA_ECOLI)	*Escherichia coli* *(strain K12)*	*ptrA*	Protease 3 (Pitrilysin)	962	7.1 × 10^−^^6^	27.6	47.6
O22941 (IDE1_ARATH)	*Arabidopsis thaliana (Mouse-ear cress)*	*PXM16*	Insulin-degrading enzyme-like 1, peroxisomal	970	0.0	38.5	57.1
F4J3D9 (IDE2_ARATH)	*Arabidopsis thaliana (Mouse-ear cress)*	*At3g57470*	Insulin-degrading enzyme-like 2	966	0.0	39.4	57.9
Q06010 (STE23_YEAST)	*Saccharomyces cerevisiae (strain ATCC 204508/S288c) (Baker’s yeast)*	*STE23*	A-factor-processing enzyme	1027	0.0	38.4	60.2
P40851 (AXL1_YEAST)	*Saccharomyces cerevisiae (strain ATCC 204508/S288c) (Baker’s yeast)*	*AXL1*	Putative protease AXL1	1208	9.2 × 10^−^^50^	21.5	41.5
P22817 (IDE_DROME)	*Drosophila melanogaster* *(Fruit fly)*	*Ide*	Insulin-degrading enzyme	990	0.0	46.6	67.3
Q9JHR7 (IDE_MOUSE)	*Mus musculus* *(House mouse)*	*Ide*	Insulin-degrading enzyme	1019	0.0	95.0	97.4
P35559 (IDE_RAT)	*Rattus norvegicus* *(Norwegian rat)*	*Ide*	Insulin-degrading enzyme	1019	0.0	95.5	97.6
Q24K02 (IDE_BOVIN)	*Bos taurus* *(Bovine)*	*Ide*	Insulin-degrading enzyme	1019	0.0	98.8	99.2
F7EFL5 (F7EFL5_MACMU)	*Macaca mulatta* *(Rhesus macaque)*	*Ide*	Insulin-degrading enzyme	1019	0.0	99.5	99.7
P14735 (IDE_HUMAN)	*Homo sapiens* *(Human)*	*Ide*	Insulin-degrading enzyme	1019	0.0	100	100

* BLAST results with P14735 (IDE_HUMAN) as query sequence.

**Table 2:** Human paralogs of IDE.

UniProtKB Entry	Organism	Gene Name	Protein Name	Protein Length	E-Value *	Identity (%) *	Positives (%) *
Q5JRX3 (PREP_HUMAN)	*Homo sapiens* *(Human)*	*PITRM1*	Presequence protease, mitochondrial	1037	7.6 × 10^−1^	28.9	51.3
Q10713 (MPPA_HUMAN)	*Homo sapiens* *(Human)*	*PMPCA*	Mitochondrial-processing peptidase subunit alpha	525	1.7 × 10^−1^	24.4	45.5
P31930 (QCR1_HUMAN)	*Homo sapiens* *(Human)*	*UQCRC1*	Cytochrome b-c1 complex subunit 1, mitochondrial	480	1.3 × 10^−2^	21.7	40.1
O75439 (MPPB_HUMAN)	*Homo sapiens* *(Human)*	*PMPCB*	Mitochondrial-processing peptidase subunit beta	489	7.9 × 10^−3^	22.8	39.6
O43847 (NRDC_HUMAN)	*Homo sapiens* *(Human)*	*NRDC*	Nardilysin	1151	8 × 10^−174^	34.6	55.6
P14735 (IDE_HUMAN)	*Homo sapiens* *(Human)*	*Ide*	Insulin-degrading enzyme	1019	0.0	100	100

* BLAST results with P14735 (IDE_HUMAN) as query sequence.

**Table 3:** Knockout mice models of hepatic insulin resistance.

Mouse Model	Genetic Background	Target Protein	Target Tissue	Metabolic Phenotype	Insulin Resistance	Insulin Clearance	Refs.
*db*/*db*	C57BLKs/J	OB-R	Spontaneous mutation of the leptin receptor	Hyperinsulinemia, hyperglycemia, higher body weight	+	n.d.	[288,289]
*ob*/*ob*	C57BLKs/J	Leptin	Recessive mutation of leptin	Hyperinsulinemia, hyperglycemia, higher body weight	+	n.d.	[289,290]
L-IDE-KO	C57BL/6J	IDE	Liver	Normoinsulinemia, higher glucose levels, similar body weight	+	=	[86,87]
LIRKO	Mixed genetic background	IR	Liver	Hyperinsulinemia, hyperglycemia, similar body weight	+	Lower	[291,292]
*LIrs1KO*	Mixed genetic background	IRS1	Liver	Hyperinsulinemia, euglycemia, similar body weight	+ (*)	n.d.	[293]
*LIrs2KO*	Mixed genetic background	IRS2	Liver	Hyperinsulinemia, euglycemia, similar body weight	+ (^$^)	n.d.	[293]
L-p110-αKO	Mixed genetic background	PI3K catalytic subunit p110-α	Liver	Hyperinsulinemia, hyperglycemia, increased fat mass	+	n.d.	[294]
L-p110βKO	Mixed genetic background	PI3K catalytic subunit p110-β	Liver	Hyperinsulinemia, similar blood glucose levels	+	n.d.	[295]
L-*Pdk1*KO	Mixed genetic background	PDK1	Liver	Hyperinsulinemia, hyperglycemia, similar body weight	+	n.d.	[296]
L-Akt2	C57BL/6J	AKT2	Liver	Normoinsulinemia, euglycemia, similar body weight	-	n.d.	[286,287]
L1KO(l-FoxO1)	C57BL/6J	FoxO1	Liver	Normoinsulinemia, lower plasma glucose, similar body weight	-	n.d.	[285,297]
LiRiKO	C57BL/6J	Rictor	Liver	Hyperinsulinemia, hyperglycemia, similar body weight	+	n.d.	[298]
L-SACC1 (*AlbCre + Cc1^fl^*^/^*^fl^*)	C57BL/6J	CEACAM1	Liver	Hyperinsulinemia, hyperglycemia, increased fat mass	+	Lower	[299]

* Insulin resistance after refeeding; ^$^ Insulin resistance during fasting; n.d., not determined; + presence of insulin resistance; - no presence of insulin resistance; = no change.
